# Leptomeningeal Carcinomatosis in Urothelial Carcinoma of the Urinary Bladder: A Report of a Patient with a Fulminant Course Who Died of Cancer after Definitive Therapies

**DOI:** 10.1155/2021/5543939

**Published:** 2021-04-30

**Authors:** Masayuki Tomioka, Makoto Kawase, Daiki Kato, Manabu Takai, Koji Iinuma, Kengo Horie, Keita Nakane, Natsuko Suzui, Tatsuhiko Miyazaki, Takuya Koie

**Affiliations:** ^1^Department of Urology, Gifu University Graduate School of Medicine, Gifu, Japan; ^2^Department of Pathology, Gifu University Graduate School of Medicine, Gifu, Japan

## Abstract

A 45-year-old Japanese man visited a community hospital with the chief complaint of asymptomatic macrohematuria. He was diagnosed with muscle-invasive bladder cancer (MIBC), and he received intra-arterial chemotherapy followed by radiation therapy at another institution. Twenty-eight months after chemoradiotherapy, magnetic resonance imaging (MRI) revealed MIBC recurrence. After neoadjuvant chemotherapy, robot-assisted radical cystectomy was performed. Pathological examination indicated high-grade urothelial carcinoma with lymphovascular invasion, a positive surgical margin, and skip lesions of cancer cells in the perivesical adipose tissue. Three months after surgery, he was brought to our hospital in an ambulance with the chief complaint of rotatory vertigo and was speaking inarticulately. Head and whole spine MRI revealed meningeal metastasis along both the vestibulocochlear nerves and cauda equina. Analysis of the cerebrospinal fluid revealed malignant cells. The patient was diagnosed with leptomeningeal carcinomatosis originating from the MIBC. He received whole-brain radiotherapy followed by the administration of pembrolizumab. Unfortunately, the patient's condition quickly deteriorated, and he died of cancer 4 months after surgery.

## 1. Introduction

Leptomeningeal carcinomatosis (LMC) is defined as malignant cell infiltration in the pia mater and arachnoid membrane [1]. Although LMC occurs in 3%–5% of cancer patients [1], only 0.03% of patients with genitourinary (GU) cancer are diagnosed with LMC [2]. In addition, only four cases of an initial diagnosis of LMC originating from bladder cancer (BC) without other metastatic sites have been reported to date [3]. Herein, we present a patient with LMC that developed at the time of the initial diagnosis of muscle-invasive BC (MIBC). The patient underwent a fulminant course and died of BC after definitive therapies.

## 2. Case Presentation

A 45-year-old Japanese man visited a community hospital with a chief complaint of asymptomatic macrohematuria. Cystoscopy revealed multiple papillary nonpedunculated tumors ranging from the left lateral wall to the anterior wall of the urinary bladder. Histopathological diagnosis revealed high-grade urothelial carcinoma (UC) of the bladder with carcinoma in situ (CIS). At this point, the patient did not exhibit lymph node involvement or distant metastases. Bladder cancer (BC) was classified as clinical T2bN0M0 according to the staging system defined in the American Joint Committee on Cancer Staging Manual [4]. Although radical cystectomy (RC) was recommended, the patient hoped to preserve the urinary bladder. The patient received one cycle of intra-arterial chemotherapy using a combination of gemcitabine and cisplatin (GC) followed by radiation therapy (36 Gy in 2 Gy fractions) for the urinary bladder at another institution [5]. Subsequently, there was no evidence of the disease.

**Figure 1:**
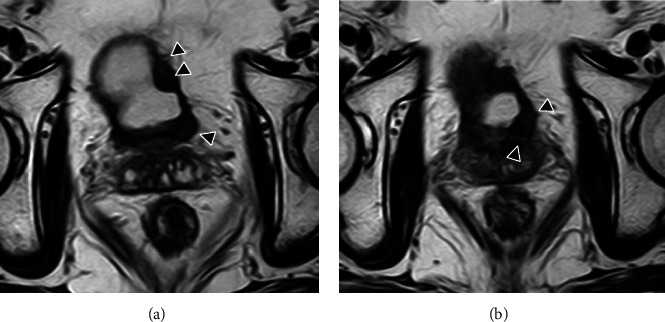
T2-weighted magnetic resonance image showing multiple recurrent tumors with muscle layer infiltration at the left laterally wall (a) and the neck (b) of the urinary bladder (arrows).

**Figure 2:**
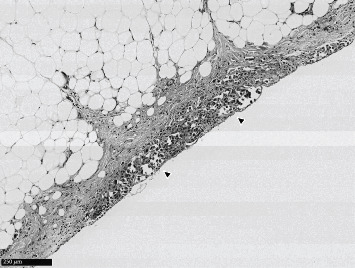
Pathological examination of the surgical specimen with skip lesions of cancer cells in the perivesical adipose tissue (arrows).

Twenty-eight months after chemoradiotherapy, magnetic resonance imaging (MRI) revealed multiple recurrent tumors with muscle layer infiltration of the left lateral wall and the neck of the urinary bladder (T2-weighted image; Figures 1(a) and 1(b)). Transurethral resection of the bladder tumor was performed, and the histopathological diagnosis was a high-grade UC with muscle layer invasion. RC was recommended again; therefore, the patient visited our hospital to undergo RC. Before surgery, he received two courses of GC (1000 mg/m^2^ gemcitabine on days 1, 8, and 15 and cisplatin 70 mg/m^2^ on day 2) as neoadjuvant chemotherapy (NAC) every 21 days [6]. Preoperative computed tomography (CT) after NAC showed that the BC was stable, and lymph node involvement and distant metastasis were not identified. Robot-assisted RC followed by intracorporeal ileal neobladder reconstruction was performed [6]. Pathological examination of the surgical specimen indicated high-grade UC, pathological stage T4a, with lymphovascular invasion, positive surgical margin, and skip lesions of the UC in the perivesical adipose tissue (Figure 2). Therefore, we decided to perform more careful observation of this patient without adjuvant chemotherapy.

**Figure 3:**
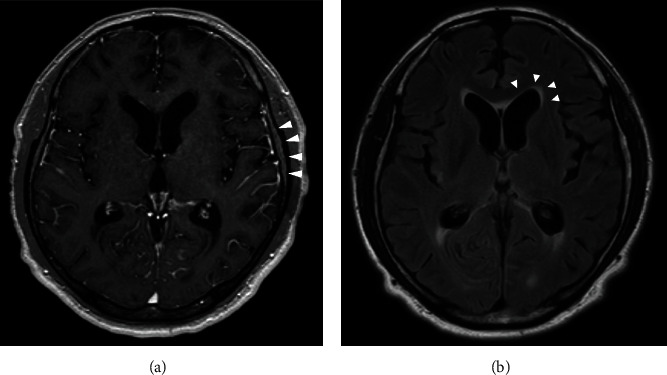
Dynamic contrast-enhanced MRI showing LMC of the cerebrum, mesencephalon, and cerebellum (a). Infiltrating tumor cells can be identified along the cerebral cortex on T2 fluid-attenuated inversion-recovery imaging (b) (arrows).

**Figure 4:**
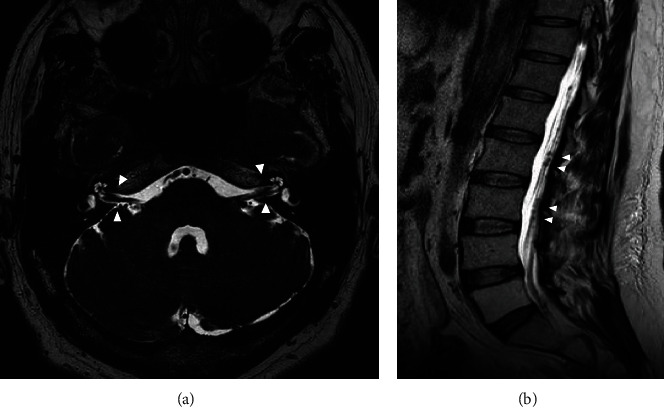
T2-weighted MRI showing vestibulocochlear nerves metastasis (a) and cauda equina metastasis (b) (arrows).

**Figure 5:**
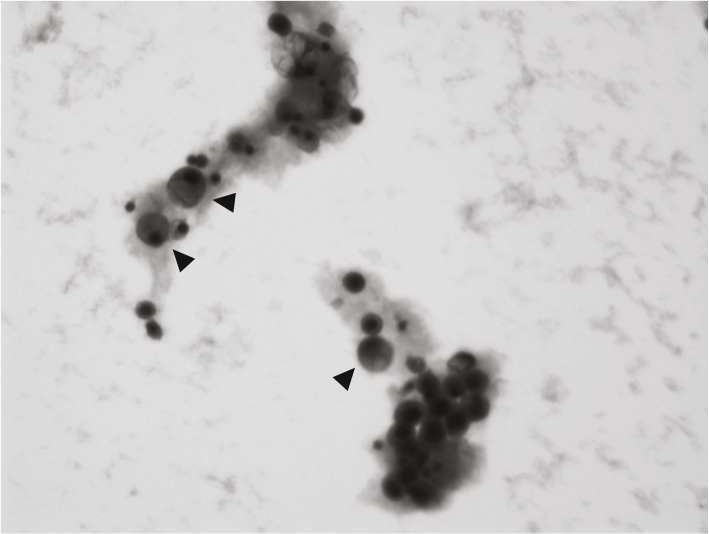
Analysis of the cerebrospinal fluid showing malignant cells (arrows).

However, he was brought to our hospital in an ambulance with the chief complaint of rotatory vertigo and speaking inarticulately 3 months after surgery. Dynamic contrast-enhanced MRI showed LMC of the cerebrum, mesencephalon, and cerebellum (Figure 3(a)). Infiltrating tumor cells were identified along the cerebral cortex on T2 fluid-attenuated inversion-recovery images (Figure 3(b)). In addition, T2-weighted MRI revealed meningeal metastasis along both the vestibulocochlear nerves and cauda equina (Figures 4(a) and 4(b)). Whole-body CT could not identify pelvic lymphadenopathy or metastasis to other organs. A lumbar puncture was performed, and analysis of the cerebrospinal fluid revealed malignant cells (Figure 5). The patient was diagnosed with LMC originating from the MIBC. Thereafter, his level of consciousness decreased without warning. Immediately, the patient was started on dexamethasone (6.6 mg) intravenously, and his level of consciousness recovered temporarily. Hence, he received whole-brain radiotherapy (30 Gy) followed by the administration of pembrolizumab (200 mg). Unfortunately, the patient's condition quickly deteriorated, and he died of BC one month after the diagnosis of LMC.

## 3. Discussion

LMC is diagnosed in 1%–5% of patients with solid tumors, 5%–15% of patients with leukemia and lymphoma, and 1%–2% of patients with primary brain tumors [7]. Autopsy studies have demonstrated that 19% of patients with cancer and neurological signs and symptoms have evidence of meningeal involvement [8]. Regarding the primary site, carcinomas of unknown origin constitute 1%–7% of all cases of LMC, even though small cell lung cancer and melanoma have the highest rates of LMC (11% and 20%, respectively) [9]. Regarding LMC originating from genitourinary cancer, in the MD Anderson Cancer Center database, only 31 (0.03%) patients were diagnosed with LMC among 93,960 patients diagnosed with GU cancer [2]. Umezawa et al. reported 33 cases of LMC originating from BC [3]. Four patients (16.7%) had already developed LMC at the initial diagnosis [3]. Conversely, 20 patients (60.6%) were diagnosed with LMC during treatment for BC, and 16 (48.5%) developed LMC without progression of the primary or metastatic sites [3]. For patients with BC who have severe systemic disease, the possible diagnosis of LMC may not have been pursued because of the overall poor prognosis of these patients [2]. Therefore, the true frequency of LMC may be difficult to estimate for patients with systemic BC or those who are frequently treated with several drugs.

LMC is usually a late manifestation of systemic disease, and most often occurs in patients after extensive therapy with surgery, radiation, and chemotherapy [10]. Tumor metastasis to the meninges typically occurs by one of four mechanisms: meningeal seeding from preexisting hemispheric central nervous system (CNS) metastases, direct extension from subdural or epidural tumors, direct extension from sites outside but adjacent to the CNS, and hematogenous spread [11]. Once cancer cells enter the subarachnoid space, they are transported by cerebrospinal fluid (CSF) flow, resulting in disseminated and multifocal neuraxis seeding of the leptomeninges [12]. In addition, tumor infiltration is most prominent at the base of the brain and on the dorsal surface of the spinal cord, especially the cauda equina [7, 13]. In our case, the nature of the metastasis to the leptomeninges remains unclear. The cause of unprecedented LMC without metastasis to regional lymph nodes or other organs suggests that the lymphatic or venous flow of the urinary bladder may have changed after receiving intra-arterial chemotherapy followed by radiation therapy for the urinary bladder before surgery.

To date, the optimal treatment for LMC remains poorly defined. For this reason, the prognosis of LMC from BC is worse, and the median survival from diagnosis to death is 35 days (interquartile range, 16–134 days) [3]. Therefore, the treatment of LMC is typically palliative and rarely curative based on four prospective randomized trials in LMC [12]. In addition, the therapeutic management strategies employed for LMC are similar to those employed for brain metastasis. Radiation therapy is used in the treatment of LMC for palliation of symptoms, decreasing bulky disease, and correcting CSF flow abnormalities [12]. However, whole neuraxis radiation is rarely indicated because it is associated with significant systemic toxicity, including severe myelosuppression and mucositis, and is not curative [12]. Conversely, systemic or intrathecal chemotherapy is a treatment modality that can medicate the entire neuraxis, multiple lymph nodes, and other organs [12]. Methotrexate (MTX) is a key drug for the treatment of LMC and is administered as a systemic and/or intrathecal chemotherapeutic agent [14]. However, most chemotherapeutic agents administered systemically have poor CSF penetration and do not reach therapeutic levels [12]. Recently, the effectiveness of molecular targeted therapy or immune checkpoint inhibitors, including bevacizumab for breast cancer, intrathecal trastuzumab for human epidermal growth factor receptor 2-positive breast cancer, and nivolumab for lung adenocarcinoma, has been reported [15–17]. It is unclear whether these drugs are effective for treating LMC originating from BC. Although pembrolizumab was used for the treatment of LMC in our case, the treatment effect was not completely achieved. It may have been better to have chosen a chemotherapy regimen, including methotrexate if possible, although the patient's condition was very poor. A promising strategy for LMC should be devised in the near future.
